# PD-L1 Blockade by Atezolizumab Downregulates Signaling Pathways Associated with Tumor Growth, Metastasis, and Hypoxia in Human Triple Negative Breast Cancer

**DOI:** 10.3390/cancers11081050

**Published:** 2019-07-25

**Authors:** Reem Saleh, Rowaida Z. Taha, Varun Sasidharan Nair, Nehad M. Alajez, Eyad Elkord

**Affiliations:** Cancer Research Center, Qatar Biomedical Research Institute (QBRI), Hamad Bin Khalifa University (HBKU), Qatar Foundation (QF), Doha P.O. Box 34110, Qatar

**Keywords:** triple negative breast cancer, anti-PD-L1, metastasis, EMT

## Abstract

Triple negative breast cancer (TNBC) is the most aggressive type of breast cancer, which shows resistance to common breast cancer therapies, as it lacks the expression of the most common breast cancer targets. Therefore, TNBC treatment remains a challenge. Targeting programmed cell death-ligand 1 (PD-L1) by monoclonal antibodies (mAbs), for example, atezolizumab, has revolutionized the treatment for various cancer types. However, the therapeutic efficacy of targeting PD-L1 in TNBC is currently under investigation. In this study, we investigated the molecular mechanisms by which the human TNBC cell line MDA-MB-231, expressing PD-L1, responds to atezolizumab, using RNA-Seq. Transcriptome analysis revealed 388 upregulated and 362 downregulated genes in response to atezolizumab treatment. The expression of selected genes, from RNA-Seq data, was subsequently validated using RT-qPCR in the MDA-MB-231 and MDA-MB-468 TNBC cells following atezolizumab treatment. Bioinformatics analysis revealed that atezolizumab downregulates genes promoting cell migration/invasion and metastasis, epithelial-mesenchymal transition (EMT), cell growth/proliferation/survival, and hypoxia. On the contrary, genes associated with apoptosis and DNA repair were upregulated in response to atezolizumab treatment. Gene set enrichment analyses revealed that a significant number of these genes are related to the NF-kB, PI3K/Akt/mTOR, MAPK, and CD40 signaling pathways. Using functional assays, we confirmed that atezolizumab increases MDA-MB-231 cell apoptosis/necrosis, and reduces their proliferation and viability. Collectively, our findings provide novel insights into the molecular mechanisms/signaling pathways by which atezolizumab exerts inhibitory effects on TNBC, thereby inhibiting EMT/metastasis, tumor growth/survival, and the induction of hypoxia.

## 1. Introduction

Breast cancer is one of the most common causes of cancer-related deaths among females around the world [1]. Amongst breast cancer subtypes, triple negative breast cancer (TNBC) is the most aggressive type of breast cancer, characterized by high grades, and high rates of distant metastasis and poor survival [2]. It constitutes 10–20% of all breast cancer cases [2]. TNBC is resistant to common breast cancer therapies, as it lacks the expression of the most common breast cancer targets, namely estrogen receptor (ER), human epidermal growth factor receptor-2 (HER2), and progesterone receptor (PR) [2,3].

Immunotherapy in the form of monoclonal antibodies (mAbs) targeting the programmed cell death 1 (PD-1)/programmed cell death-ligand 1 (PD-L1) axis have revolutionized the treatment for various cancer types [4,5,6,7,8]. Unlike these cancer types, most breast tumors are less immunogenic and exhibit low T cell infiltrate [9]. However, TNBC exhibits a high number of intratumoral and stromal tumor-infiltrating lymphocytes (TILs), suggesting that immunotherapy could be beneficial [9].

PD-L1 is primarily expressed on tumor cells, and it can be expressed by some subsets of activated myeloid cells [10]. It binds to the co-inhibitory receptor/immune checkpoint, PD-1, which is expressed by activated T and B cells [10]. The interaction between PD-1 and PD-L1 halts the activation of cytotoxic T cells, induces T cell apoptosis, and reduces the production of cytotoxic cytokines, thereby suppressing anti-tumor immune responses [11]. Despite the fact that the therapeutic efficacy of targeting the PD-1/PD-L1 axis has been shown in patients with melanoma, non-small cell lung cancer (NSCLC), and renal cell carcinoma [12,13], there is accumulating evidence suggesting that PD-L1 can facilitate tumor resistance, and promote tumor growth and survival against traditional cancer therapies and immunotherapy [14,15]. Other studies reported that PD-L1 increases tumor cell resistance to pro-apoptotic signals induced by cytotoxic T cells or chemotherapeutic and radiotherapeutic agents [16,17]. In line with this, it has been demonstrated that upregulated expression of PD-L1 on cancer cells, in response to DNA damage, is associated with the development of tumor resistance to cell death [17,18]. Moreover, PD-L1 has been implicated in the induction of epithelial-mesenchymal transition (EMT) in esophageal cancer [19], a process that is responsible for the conversion of a benign tumor to a highly invasive metastatic tumor [20].

In breast cancer patients, the expression of PD-L1 shows a positive correlation with the number of TILs, histological grades, and poor overall survival rates [21,22]. In addition, PD-L1 expression has been detected in approximately 20–30% of TNBC cases [23,24,25]. These findings prompted the evaluation of the clinical efficacy of targeting PD-L1 in breast cancer patients, including those with TNBC [9,26]. However, the mechanisms by which anti-PD-L1 mAb exerts therapeutic effects on breast cancer cells have not been fully elucidated. Therefore, it is important to identify the mechanisms and signaling pathways affected by anti-PD-L1 mAb, and to understand how it alters the function and molecular profile of breast cancer cells.

In this study, we sought to identify the mechanisms by which human TNBC cells expressing PD-L1 are affected by anti-PD-L1 mAb treatment using atezolizumab. We utilized the TNBC cell line MDA-MB-231, as it expresses high levels of PD-L1 [27,28]. We examined the effects of atezolizumab on the expression of PD-L1 at the gene and protein levels. Next, we performed RNA-Seq analysis to determine the effects of atezolizumab on the whole transcriptome of MDA-MB-231.

Our results show that atezolizumab blocks the surface expression of PD-L1 without altering PD-L1 expression at the mRNA and protein levels. Moreover, we revealed some of the molecular mechanisms/pathways by which atezolizumab induces anti-tumor effects and alters the function and molecular profile of the highly aggressive TNBC cell line, MDA-MB-231. Using RNA-Seq analysis, we found that atezolizumab negatively regulates tumor growth/metastasis, EMT, DNA damage, and hypoxia. We also confirmed the RNA-Seq data by performing functional analyses and determined the effect of atezolizumab on MDA-MB-231 cells using cell death/apoptosis, proliferation, and viability assays. We showed that atezolizumab increases necrosis/apoptosis, and reduces the proliferation and viability of MDA-MB-231 cells.

## 2. Results

### 2.1. Atezolizumab Blocks the Epitope of PD-L1 and Does Not Alter PD-L1 mRNA and Protein Expression

**Figure 1:**
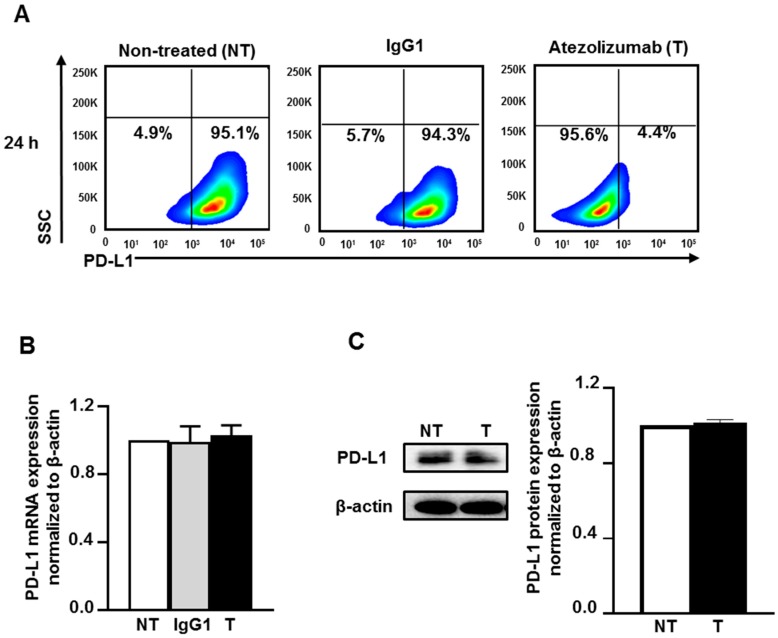
Effect of atezolizumab on PD-L1 expression in MDA-MB-231 cells. MDA-MB-231 cells were cultured without or with 0.5 µg/mL of human IgG1 (isotype control) or atezolizumab. The surface expression of PD-L1 was determined at 24 h post treatment by flow cytometry. Representative flow cytometric plots show the percentage of cells expressing PD-L1 in non-treated, IgG1- and atezolizumab-treated cells (**A**). The expression of PD-L1 mRNA was determined by RT-qPCR (**B**). The expression of PD-L1 protein was determined by western blot and densitometeric analysis (**C**). More detailed western blot information can be found in Supplementary Materials (Figure S1). The relative expression of mRNA and protein were normalized to β-actin. Results are from two independent experiments, and expressed as the mean ± SEM.

To address the mechanisms of the action of atezolizumab in MDA-MB-231, we first examined the effect of atezolizumab on PD-L1 (protein and mRNA) expression. The surface expression of PD-L1 was determined by flow cytometric analysis. Almost all MDA-MB-231 cells (non-treated or treated with human IgG1 isotype control) were positive for PD-L1, however the detection of the PD-L1 epitope was blocked by the specific antibody, atezolizumab, after 24 h treatment (Figure 1A). RT-qPCR gene expression analysis showed that the PD-L1 mRNA expression level was not affected upon atezolizumab treatment, compared to non-treated cells and cells treated with isotype control (Figure 1B). The expression of PD-L1 mRNA level in the isotype-treated cells was similar to that of non-treated cells (Figure 1B). Additionally, data from western blots showed no difference in PD-L1 protein expression following atezolizumab treatment at 24 h (Figure 1C).

Collectively, these data indicate that atezolizumab blocks the epitope of PD-L1 without altering the expression of PD-L1 at both the mRNA and protein levels. It is most likely that atezolizumab blocks the detection of PD-L1 by the anti-PD-L1-APC antibody used for flow cytometry. Atezolizumab binding to PD-L1 at the cell surface would hamper the binding of the anti-PD-L1-APC antibody. It is worth noting that the isotype control has no effect on the epitope of PD-L1, suggesting the specificity of atezolizumab in blocking PD-L1 antigen.

### 2.2. Atezolizumab Downregulates Genes Promoting Cell Migration/Metastasis and EMT

We then investigated the molecular mechanisms by which atezolizumab exerts its anti-tumor effects on MDA-MB-231 cells. At 24 h, RNA from treated and non-treated cells was extracted, and RNA-Seq was performed.

**Figure 2:**
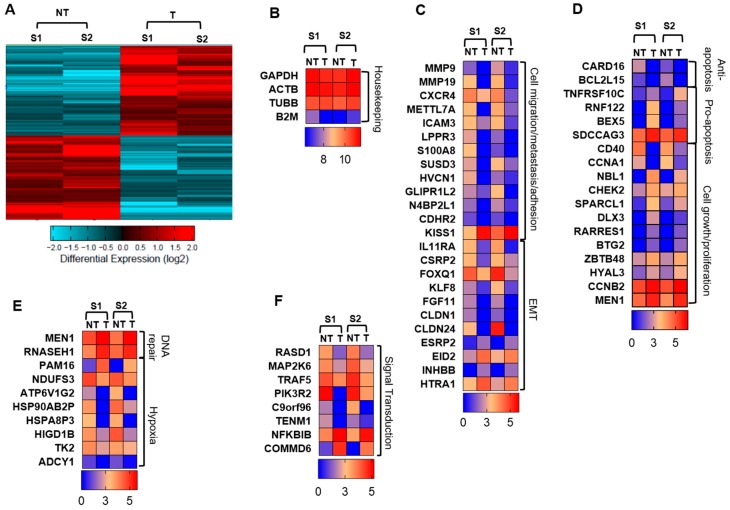
Differentially expressed genes in MDA-MB-231 cells following atezolizumab treatment. Hierarchical clustering of two independent experiments on differentially expressed RNA transcripts from RNA-Seq data. Each column represents a sample and each row represents a transcript. Expression level of each gene in a single sample is depicted according to color scale (**A**). Heat maps show the fold changes relative to the mean expression of housekeeping genes (**B**), cell migration/metastasis/adhesion and EMT (**C**), anti-apoptosis, pro-apoptosis, and cell growth/proliferation (**D**), DNA repair and hypoxia (**E**), and signaling transduction (**F**). Results are from two independent experiments. S1 = sample 1; S2 = sample 2; NT = non-treated cells; T = treated cells with atezolizumab.

The hierarchal clustering shows a distinct cluster of genes which are differentially regulated in the treated cells, compared with non-treated cells (Figure 2A). We found that atezolizumab treatment upregulated 388 genes and downregulated 362 genes in MDA-MB-231 cells. Genes from RNA-Seq data were identified and classified into categories using GeneCards, Uniprot gene, protein databases and the literature. Heat maps for fold change in different genes expressed in two samples (S1 and S2) of either non-treated cells or treated cells are shown in Figure 2B–D. The quality of data was confirmed using the fold changes in housekeeping genes, including *GAPDH, ACTB, TUBB*, and *B2M*, which were similar in non-treated and treated cells (Figure 2B). Interestingly, genes promoting cell migration/invasion and metastasis, including *MMP9, MMP19, CXCR4, METTL7A, ICAM3, LPPR3, S100A8, SUSD3, HVCN1, GLIPR1L2, N4BP2L1* and *CDHR2*, were significantly downregulated in MDA-MB-231 cells treated with atezolizumab, compared with non-treated cells (*p* < 0.05, Figure 2C). *KISS1* gene, metastasis suppressor gene, was upregulated in MDA-MB-231 cells treated with atezolizumab, compared with non-treated cells (*p* < 0.05, Figure 2C). Additionally, genes favoring EMT were significantly downregulated upon atezolizumab treatment, such as *IL11RA, CSRP2, FOXQ1, KLF8, FGF11, CLDN1*, and *CLDN24* (*p* < 0.05, Figure 2C). Genes that inhibit EMT were upregulated following atezolizumab treatment, such as *ESRP2, EID2, INHBB,* and *HTRA1* (*p* < 0.05, Figure 2C).

### 2.3. Atezolizumab Downregulates Anti-Apoptotic Genes, Upregulates Pro-Apoptotic Genes, and Downregulates Genes Involved in Cell Growth and Proliferation

Our data from RNA-Seq analysis showed that anti-apoptotic genes, *CARD16* and *BCL2L15*, were significantly downregulated following atezolizumab treatment (*p* < 0.05, Figure 2D), while pro-apoptotic genes, *TNFRSF10C, RNF122, BEX5, SDCCAG3*, were significantly upregulated in MDA-MB-231 cells treated with atezolizumab (*p* < 0.05, Figure 2D). Genes favoring tumor growth and cell proliferation, *CD40* and *CCNA1* were significantly downregulated in treated cells (*p* < 0.05, Figure 2D). On the other hand, tumor suppressor genes and genes inhibiting cell growth, such as *CHEK2*, *SPARCL1*, *DLX3*, *RARRES1*, *NBL1*, *CCNB2*, *FGF18*, *HYAL3*, *BTG2*, *ZBTB48*, and *MEN1*, were upregulated in treated cells (*p* < 0.05, Figure 2D).

### 2.4. Atezolizumab Upregulates DNA Repair Genes and Downregulates Genes Related to Hypoxia

The involvement of PD-L1 with DNA repair, genomic instability, and hypoxia has not been extensively investigated. Here, we show that genes associated with DNA repair, *MEN1* [29], and *RNASEH1* [30], were upregulated following atezolizumab treatment (*p* < 0.05. Figure 2E). Additionally, atezolizumab upregulated the *PAM16* gene, which could act as a tumor suppressor gene and a regulator of ATP synthesis (*p* < 0.01, Figure 2E). Notably, genes related to hypoxia/ATP synthesis or genes encoding heat shock proteins, including *NDUFS3*, *ATP6V1G2*, *HSP90AB2P*, *HSPA8P3*, *HIGD1B*, *TK2*, and *ADCY1* (*p* < 0.05, Figure 2E), and also genes associated with the PI3K, MAPK, and NF-kB signaling pathways, including *RASD1*, *MAP2K6*, *TRAF5*, *PIK3R2*, *C9orf96*, and *TENM1*, were significantly downregulated in treated cells (*p* < 0.05, Figure 2F). In contrast, inhibitors for NF-kB activation, *NFKBIB* and *COMMD6*, were significantly up-regulated upon atezolizumab treatment (*p* < 0.05, Figure 2F).

### 2.5. Atezolizumab Downregulates NF-kB, Akt, and CD40 Signaling Pathways

**Figure 3:**
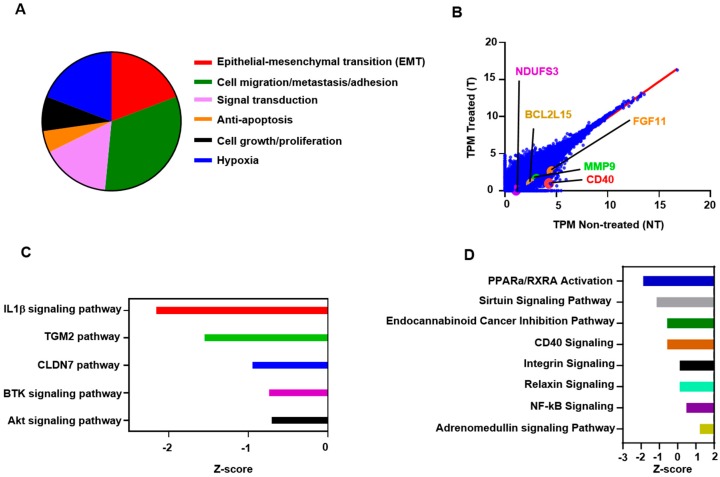
Multiple affected canonical and signaling pathways in MDA-MB-231 cells in response to atezolizumab treatment. A pie-chart shows the proportion of genes that were downregulated post atezolizumab treatment (**A**). RNA-Seq analyses in MDA-MB-231 are represented by scatter plots. *X* and *Y* axes showing Log2 TPM (transcripts per million) of non-treated and treated cells (**B**). Top significantly affected (−0.5 < *Z* score < −2.5) pathways based on the upstream regulator analysis (URA). The horizontal bars denote the different pathways based on the *Z*-scores (**C**). Top significantly affected (1.5 < *Z* score < −2.0) canonical pathways based on ingenuity pathway analysis (IPA). The horizontal bars denote the different pathways based on the *Z*-scores (**D**). Results are from two independent experiments.

Next, we found that about 19% of the genes which were downregulated in atezolizumab-treated cells are associated with EMT, 33% are related to cell migration/invasion and metastasis, 16% are associated with signaling transduction, favoring cell proliferation and EMT, 5% are anti-apoptotic, 8% are related to cell growth and tumor cell proliferation, and 19% are associated with hypoxia (Figure 3A). Selected genes from both upregulated and downregulated panels, including *CD40, MMP9, BCL2L15*, and *NDUFS3*, are shown in Figure 3B—values below the linear regression line (as shown in red) indicate a “downregulation” and above the line indicate an “upregulation”.

Based on the upstream regulator analysis (URA), the effect of atezolizumab on MDA-MB-231 cells is significantly related to several functional networks and signaling pathways (Figure 3C). These pathways have been implicated either in cell proliferation/growth/migration and invasion, metabolism, or apoptosis, such as AKT [31], BTK (Bruton’s tyrosine kinase) [32], CLDN7 (Cluadin-7) [33], TGM2 (Tissue transglutaminase pathway) [34], and IL1β (Interleukin-1β) [35,36] (−0.5 < *Z* score < −2.5, Figure 3C). Using ingenuity pathway analysis (IPA), we showed that atezolizumab regulates other signaling pathways, such as peroxisome proliferator-activated receptor alpha (PPARa)/retinoid X receptor a (RXRa) activation, the sirtuin signaling pathway, the endocannaboid cancer inhibition pathway, the CD40 signaling pathway, the integrin pathway, the relaxin pathway, and the NF-kB and adrenomedullin signaling pathways (1.5 < *Z* score < −2.0, Figure 3D).

Together, these data indicate that atezolizumab in MDA-MB-231 cells is able to downregulate genes and signaling pathways, favoring cell migration/invasion/metastasis, EMT, tumor growth/survival, and genes related to hypoxia. In addition, our data suggest that atezolizumab may modulate functional regulatory networks related to several signaling pathways, such as the sirtuin, CD40, and NF-kB pathways.

### 2.6. Functional and Network Analyses Identified Key Genes Associated with the Response of MDA-MB-231 to Atezolizumab

**Figure 4:**
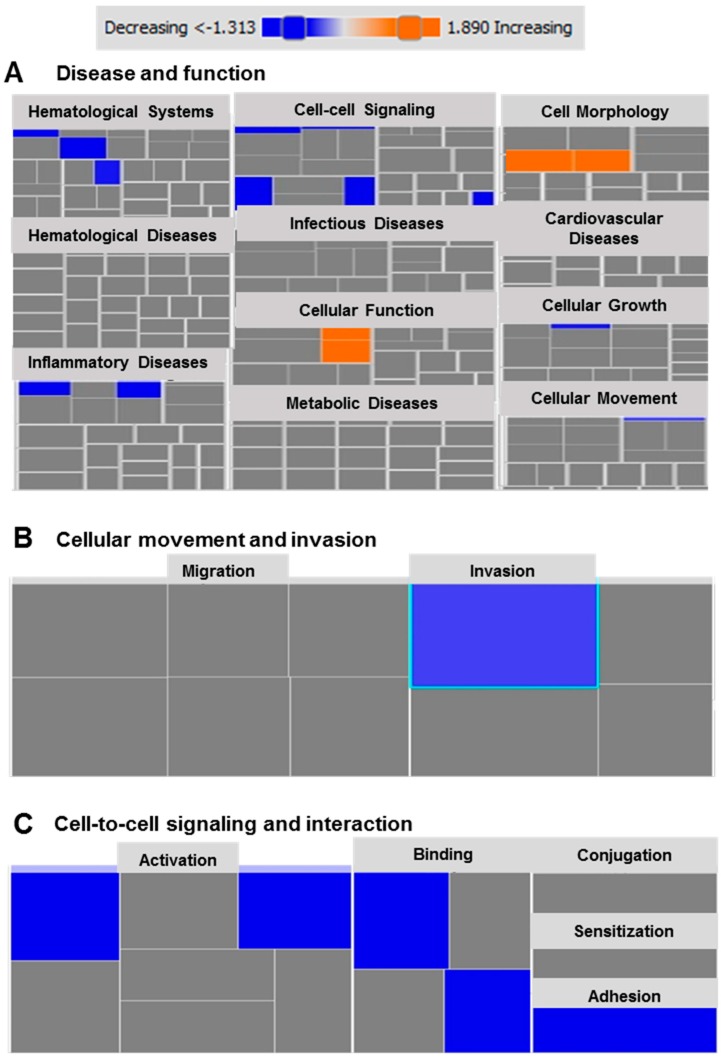
Ingenuity Pathways Analysis for differentially expressed genes in MDA-MB-231 cells following atezolizumab treatment. Tree map (hierarchical heat map) depicting affected functional categories based on differentially-expressed transcripts in response to atezolizumab treatment, where the major boxes represent a category of disease and functions (**A**). Each individual colored rectangle is a particular biological function and the color range indicates its predicted activation state: increasing (orange), or decreasing (blue). In this default view, the size of the rectangle is correlated with increased overlap significance. Tree map depicting affected functional categories based on the down-regulated transcripts in cell movement and invasion (**B**), and cell-to cell signaling and interaction (**C**).

Next, we performed gene set enrichment analysis using the Ingenuity Pathway Analysis (IPA) tool on the differentially-expressed genes in MDA-MB-231 cells in response to atezolizumab. Based on our analysis, we identified several disease-associated or function-associated pathways (Figure 4A). Importantly, we found that cellular movement and invasion (*p* < 0.01, Figure 4B), and cell-to-cell signaling and interaction (*p* < 0.05, Figure 4C) are the most suppressed functional categories. Using the KEGG (Kyoto Encyclopedia of Genes and Genomes) pathway analysis of upregulated genes, we found that the effect of atezolizumab treatment is associated with genes related to the P53 pathway, indicating the potential functions of atezolizumab in MDA-MB-231 cells, including cell cycle arrest, apoptosis, inhibition of angiogenesis and metastasis, DNA repair and damage prevention, and inhibition of the mTOR signaling pathway (Figure S2). These KEGG pathway findings are in agreement with our data from the RNA-Seq analysis.

### 2.7. Validation of RNA-Seq Data by RT-qPCR

**Figure 5:**
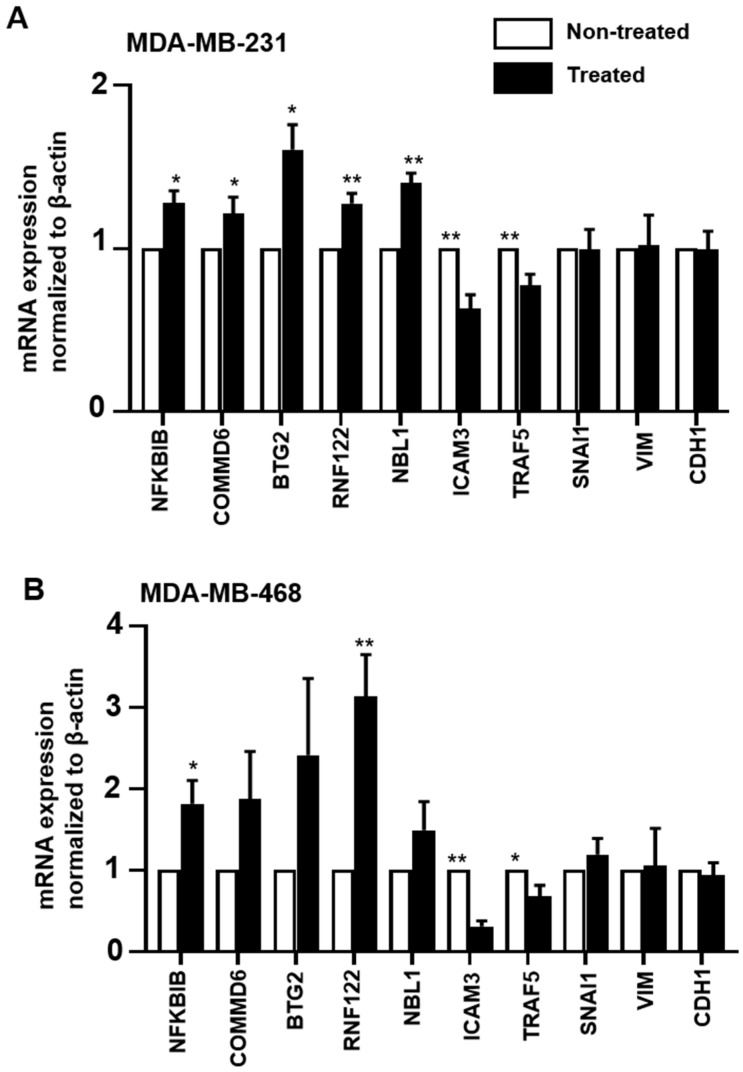
RT-qPCR gene validation in MDA-MB-231 and MDA-MB-468 cells. MDA-MB-231 and MDA-MB-468 cells were cultured without or with 0.5 µg/mL of atezolizumab for 24 h. The mRNA expression levels were determined by RT-qPCR in MDA-MB231 (**A**) and MDA-MB-468 cells (**B**). The relative gene expression was normalized to *β-ACTIN*. Results are from two experiments (three–six replicates). Paired *t*-test, * *p* < 0.05, ** *p* < 0.01.

A select number of upregulated (*NFKBIB*, *COMMD6*, *BTG2*, *RNF122*, and *NBL1*), downregulated (*IFITM10*, *ICAM3*, and *TRAF5*) and unaffected (*SNAI1*, *VIM*, and *CDH1*) genes from the RNA-Seq data were subsequently validated using RT-qPCR. We also validated these genes in another TNBC cell line, MDA-MB-468, to determine whether atezolizumab affects the tested genes in the two TNBC cell lines in a similar manner. We found that the results from RT-qPCR were consistent with RNA-Seq in MDA-MB-231 cells (Figure 5A). The same genes showed a similar pattern of expression in MDA-MB-468 cells (Figure 5B).

### 2.8. Atezolizumab Increases Necrosis/Apoptosis and Reduces Proliferation and Viability in MDA-MB-231 Cells

**Figure 6:**
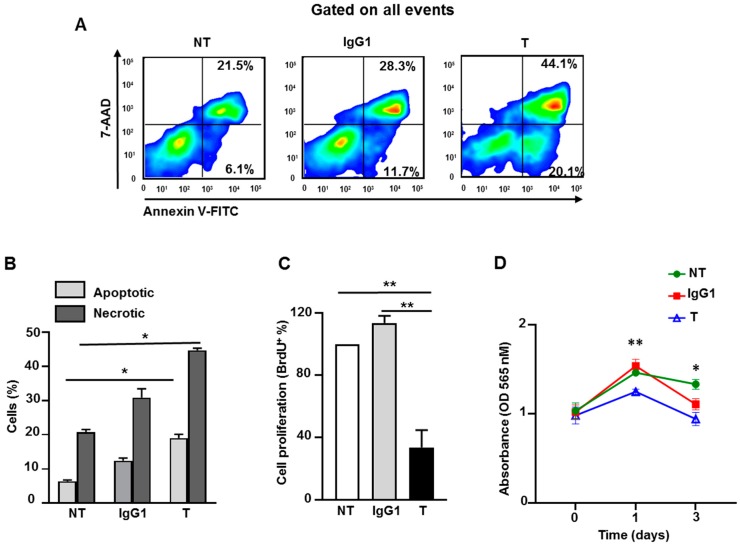
Effect of atezolizumab on MDA-MB-231 cell death/apoptosis, proliferation, and viability. MDA-MB-231 cells were cultured without or with 0.5 µg/mL of human IgG1 (isotype control) or atezolizumab for five days. Representative flow cytometric plots show the percentage of apoptotic cells and necrotic cells (**A**). Overall percentages of apoptotic and necrotic cells (non-treated (NT), IgG1- or atezolizumab-treated cells (T)) (**B**). Percentage in cell proliferation using a BrdU assay (**C**). Absorbance readings for MTT in MDA-MB-231 cells (**D**). Results are from two independent experiments (three–six replicates), and they are expressed as the mean ± SEM. Paired *t*-test, * *p* < 0.05, ** *p* < 0.01.

Next, we investigated the in vitro effect of atezolizumab on MDA-MB-231 cells using death/apoptosis and cell proliferation assays. On day six following treatment, using Annexin V and the 7-AAD detection kit, we found that atezolizumab increases the proportion of apoptotic (Annexin V^+^) and necrotic cells (Annexin V^+^ 7-AAD^+^), compared to non-treated cells or those treated with IgG1 (Figure 6A,B). We performed a BrdU (bromodeoxyuridine/5-bromo-2′-deoxyuridine) assay to detect BrdU incorporation into cellular DNA during cell proliferation, and to determine the effect of atezolizumab on MDA-MB-231 cell proliferation. On day six after treatment, results show that atezolizumab reduced MDA-MB-231 proliferation, compared to no treatment or IgG1 controls (Figure 6C). Additionally, we found that atezolizumab reduced MDA-MB-231 cell viability using MTT (3-(4,5-dimethylthiazol-2-yl)-2,5-diphenyltetrazolium bromide) assay (Figure 6D).

## 3. Discussion

The efficacy of targeting PD-1/PD-L1 axis in TNBC is still under clinical investigation, however, early phase clinical trials are showing some promising results (as reviewed in [9]). The positive association between PD-L1 expression and EMT [20,37], in addition to cancer metastasis and resistance to apoptosis [17,18,28], has been established. However, the effect of its blockade on TNBC is still to be elucidated. Therefore, it is vital to understand the underlying mechanisms by which anti-PD-L1 mAb exerts its effects, and the signaling pathways it regulates to induce anti-tumor responses in TNBC.

For a signal transduction, an interaction between ligand and receptor is usually required. In this study, we suggest that atezolizumab can alter the transcription profile of TNBC cells, MDA-MB-231, independently of the PD-1/PD-L1 interaction. Our findings could imply that the changes in MDA-MB-231 transcriptome in response to atezolizumab treatment are not due to a change in PD-L1 expression. However, this could occur as a result of an altered PD-L1 protein structure [38]. Consistent with this, our recent work showed that atezolizumab treatment alters the structure of PD-L1 on MDA-MB-231 cells by inducing a transition from a random coil and α-helical structure to β-sheet conformation [39]. Independently of the PD-1/PD-L1 signaling pathway, the intracytoplasmic domain of PD-L1 was shown to be functional [16,40,41], therefore, alterations in PD-L1 protein structure by atezolizumab might have a negative impact on its function.

We showed that the blockade of PD-L1 in MDA-MB-231 downregulates genes and signaling pathways associated with tumor aggressiveness, which is consistent with other findings demonstrating the direct role of PD-L1 in promoting tumor cell proliferation and survival [42,43]. We found 62 genes to be differentially regulated in MDA-MB-231 cells upon atezolizumab treatment. A large proportion of the downregulated genes were related to EMT, cell migration/invasion and metastasis, cell proliferation/growth, and anti-apoptosis. In support of this, the relationship between PD-L1 expression and EMT induction was previously shown in esophageal squamous cell carcinoma (ESCC) [20] and in gastric and pancreatic cancer [21]. The knockdown of PD-L1, using short interfering RNA, in esophageal cancer cell lines was shown to be effective in reducing EMT [20]. Unlike these cancer cell lines [20], the inhibition of PD-L1 in MDA-MB-231 did not affect the snail/vimentin-induced EMT pathway. This suggests that PD-L1 can regulate different pathways to induce EMT and tumor invasion in a manner that could be specific to tumor type. Thar Min et al. showed that EMT-converted tumor cells (esophageal cancer cell lines) expressing high levels of PD-L1 are able to induce T cell apoptosis using co-culture systems [37]. Other studies showed that PD-L1 can act as a pro-survival factor for tumor cells and protect tumor cells from apoptotic signals induced by T cells, cytokine-induced killer cells (CIK) therapy, chemotherapy, or radiotherapy [6,16,43].

The increase in PD-L1 expression has been reported in various types of cancer cell lines in response to DNA double-strand break (DBS) [44] or DNA damage induced by ionizing radiation and cisplatin [18]. It was reported that PD-L1 expression is regulated by Rad3-related (ATR) kinase, and that the inhibition of ATR downregulates PD-L1 and increases the sensitivity of MDA-MB-231 cells to cytotoxic T cells, killing using co-culture systems [18]. Combining the latter finding with our RNA-Seq data, which indicate that the blockade of PD-L1 upregulates genes associated with DNA repair, such as *MEN1* [29] and *RNASEH1* [30], suggests the involvement of atezolizumab in regulating DNA damage response/genomic instability and telomere integrity in cancer cells. In addition, *MEN1* could act as a tumor suppressor gene by regulating the mTOR signaling pathway (a pathway that has been implicated in cancer progression [45]) to restore CD8^+^ T cell activity and function [46]. Our RNA-Seq data analyses showed that atezolizumab significantly downregulates genes related to hypoxia. The role of hypoxia or hypoxic stress within the tumor microenvironment (TME) has been established in cancer progression—hypoxia leads to epigenetic modification, genomic instability, altered metabolism, tumor plasticity/heterogeneity and metastasis, EMT, immunosuppression, and anti-tumor immune system evasion [47]. In support of this, our IPA analysis showed that the sirtuin signaling pathway, a pathway which has been implicated in metabolism, genomic instability, and epigenetic modification [48,49], was downregulated upon atezolizumab treatment. On these bases, we conclude that the negative regulation of hypoxia/hypoxic stress could be one of the mechanisms by which atezolizumab exerts an anti-tumor effect in TNBC, suggesting a therapeutic potential for targeting PD-L1 in regulating DNA damage.

Data from our URA analyses pointed out that PD-L1 function is related to several functional regulatory networks and signaling pathways. Of interest, our analyses indicated that Akt signaling could act upstream of *MMP9* gene expression, and that the IL1β signaling pathway could act upstream of the CD40 and NF-kB signaling pathways. The association between the CD40 and NF-kB signaling pathways and PD-L1 function was highlighted by the downregulation of genes encoding components of CD40 and NF-kB signaling pathways following atezolizumab treatment. The functions of these genes, namely *CD40* [50], *MAP2K6* [51], *TRAF5* [52], *PI3K2R* [53], have been reported in enhancing cell proliferation and tumor growth/metastasis. Therefore, these findings highlight the relationship between PD-L1 and the NF-kB, PI3K/Akt, MAPK and CD40 signaling pathways. These pathways are interrelated and share common downstream mediators, for example, the cross-talk between the PI3K/Akt and NF-kB signaling pathways has been implicated in lymphoma progression by enhancing anti-apoptosis and promoting tumor growth and cell proliferation [54,55]. In addition, the role of the MAPK signaling pathway in promoting EMT, cancer cell growth and migration, and anti-apoptosis is well-established [51,56]. Collectively, these data show the involvement of PD-L1 in regulating these signaling pathways and their associated cellular and molecular events. In addition, our functional results confirm the RNA-Seq data and showed that atezolizumab increases necrosis/apoptosis, and reduces the proliferation and viability of MDA-MB-231 cells.

## 4. Materials and Methods

### 4.1. Cell Culture

Human TNBC cell lines, MDA-MB-231 and MDA-MB-468, from American Type Culture Collection (ATCC, Gaithersburg, MD, USA) were authenticated by Short Tandem Repeat (STR) Fingerprinting at the Regional Facility for DNA Fingerprinting, Rajiv Gandhi Centre for Biotechnology, India. Cells were maintained in RPMI-1640 medium (Life Technologies, New York, NY, USA), supplemented with 10% fetal calf serum (FCS) (Hyclone, GE Healthcare Life Sciences, Logan, UT, USA) and 1% Penicillin/Streptomycin (Hyclone) in a humidified incubator at 37 °C in 5% CO_2_. A total of 1 × 10^6^ cells per well were plated in a 12-well tissue culture-treated plate. Cells were cultured untreated or treated with 0.5 µg/mL of human IgG1 (isotype control, Sigma-Aldrich, St. Louis, MO, USA) or anti-PD-L1 mAb (atezolizumab, BioVision Inc., Milpitas, CA, USA). Cells were harvested at 24 h post-treatment for cell surface staining, and for RNA and protein extraction.

### 4.2. Flow Cytometry

Flow cytometric analysis was used to determine the cell surface expression of PD-L1 on the MDA-MB-231 cell line following atezolizumab treatment. Cells were trypsinized with 0.25% trypsin (Life Technologies) and collected in a complete medium. Cells were washed in phosphate-buffered saline (PBS), re-suspended, and stained with anti-PD-L1-Allophycocyanin (APC) (Clone MIH1; BD Pharmingen, San Diego, CA, USA) and cell viability dye 7-AAD (BioLegend, San Diego, CA, USA) for 30 min. Cells were washed in staining buffer (PBS with 2% FCS and 0.1% sodium azide), and re-suspended in flow cytometry (FACS) staining buffer (eBioscience, San Diego, CA, USA). Data were acquired by a BD LSRFortessa X-20 flow cytometer (BD Biosciences, San Jose, CA, USA), and analyzed by FlowJo v.10.0 software (Tree Star, Ashland, OR, USA).

### 4.3. Western Blot

MDA-MB-231 cells, non-treated or treated with atezolizumab, were lysed with 1× RIPA lysis buffer (Thermo Fisher Scientific, Waltham, MA, USA) containing proteinase inhibitor (Sigma-Aldrich) for 1 h on ice, with a vigorous vortexing every 10 min. Lysates were then centrifuged at 14,000 rpm at 4 °C for 15 min. Supernatant was collected and protein concentration was quantified using the Pierce^TM^ BCA Protein Assay Kit (Thermo Fisher Scientific). An equal amount of protein sample was loaded into NuPAGE 4–12% of Bis-Tris gel (Life Technologies Corporation, Carlsbad, CA, USA), separated by SDS-PAGE at 110 V for 1 h, and transferred onto nitrocellulose membranes (0.45 µm; Amersham Biosciences, Little Chalfont, UK). Membranes were blocked in 5% skim dry milk (Sigma-Aldrich) in 1× Tris-buffered saline with 0.1% Tween 20 detergent (TBST) buffer (Sigma-Aldrich) for 1 h at room temperature. Following three washes with 1× TBST, membranes were incubated with primary antibodies (1:1000 dilution), including anti-β-actin and anti-PD-L1 (Cell Signaling Technology, Danvers, MA, USA), in 5% skim dry milk with 1× TBST buffer for overnight at 4 °C. Following three washes with 1× TBST, membranes were incubated with an HRP-conjugated anti-rabbit secondary antibody (Abcam, 1:15,000 dilution) for 1 h at room temperature. Bands were visualized using the SuperSignal^TM^ West Pico PLUS Chemiluminescent Substrate Kit (Thermo Fisher Scientific). Blot images were acquired by Molecular Imager^®^ ChemiDoc^TM^ XRS+ with image Lab^TM^ Software (Bio-rad, Hercules, CA, USA). ImageJ software (National Institutes of Health, Bethesda, MD, USA) was used for the densitometric analysis.

### 4.4. RNA Extraction and Reverse Transcription

Total RNA was extracted and purified from MDA-MB-231 cells, non-treated and treated, using the Monrach Total RNA Miniprep Kit (New England Biolabs, Ipswich, MA, USA). RNA concentration and purity were determined by NanoDrop 2000c spectrometer (Thermo Fisher Scientific). RNA was reverse transcribed into cDNA using QuantiTect Reverse Transcription Kit (Qiagen, Hilden, Germany).

### 4.5. Real-Time Quantitative Reverse Transcriptase Polymerase Chain Reaction

The real-time RT-qPCR was performed using QuantStudio 6/7 Flex Real-time PCR system (Applied Biosystems, California, USA) for *β-ACTIN*, *PD-L1*, *NFKBIB*, *COMMD6*, *BTG2*, *RNF122*, *NBL1*, *ICAM3*, *TRAF5*, *SNAI1*, *VIM*, and *CDH1* (Integrated DNA Technologies, Inc., Coralville, IA, USA) with PowerUp SYBR Green Master Mix (Applied Biosystems). All samples were assayed in duplicate. Quantification of relative gene expression was determined, using 2^−ΔΔCT^, and normalized to β-actin. Sequences for the primers are shown in Table 1.

### 4.6. RNA Library Preparation and RNA-Seq

The quality of RNA was evaluated by the Agilent 2100 Bioanalyzer (Agilent Technologies, Santa Clara, CA, USA) with the RNA 6000 Nano LabChip Kit. Samples with RNA integrity number (RIN) greater than 7 were used for library preparation. RNA was quantified by Qubit fluorometer and the Qubit RNA broad range (BR) assay Kit (Thermo Fisher Scientific). For library preparation, 100 ng of RNA and Illumina^®^ TruSeq Stranded RNA Library Preparation Kit (Illumina, Foster City, CA, USA) were used. Briefly, RNA was purified using poly-oligo beads, and fragmented into small pieces under high temperature by divalent cation. Using random hexamers, fragments of RNA were reverse transcribed to first-stranded cDNA. The second strand of cDNA was synthesized by incorporating dUTP instead of dTTP. The adaptors were then ligated to the double-stranded cDNA, followed by a single “A” nucleotide adenylation at 3′ end of blunt fragments. The Qubit dsDNA high sensitivity (HS) assay kit (Thermo Fisher Scientific) was used to determine the yield of DNA libraries, and Agilent 2100 Bioanalyzer DNA1000 chip (Agilent Technologies) was used to determine the size distribution of the DNA libraries. The clusters were generated by cBot, and sequencing was done on a HiSeq 4000 system with 300 bp paired-end, using the HiSeq 3000/4000 SBS Kit (Illumina).

### 4.7. RNA-Seq Analysis

Paired-end reads were aligned to the hg19 human reference genome in CLC Genomics Workbench 12 (Qiagen). The abundance of transcripts was expressed as a score of TPM (transcripts per million), and mapped reads in CLC Genomics Workbench 12. Abundance data were subsequently subjected to differential gene expression and clustering analyses using 2.0-fold change and a <0.05 *p*-value cut-off.

### 4.8. Gene Set Enrichment Analyses and Modeling of Gene Interaction

Differentially-expressed genes were imported into the Ingenuity Pathways Analysis (IPA) Software (Ingenuity Systems Inc., New York, NY, USA). Functional regulatory networks and canonical pathways were determined using upstream regulator analysis (URA), downstream effects analysis (DEA), mechanistic networks (MN), and casual network analysis (CNA) prediction algorithms. IPA uses precise database to paradigm functional regulatory networks from a list of individual genes and determines a statistical score, the *Z*-score, for each network, according to the fit of the network to the set of focus genes. The biological functions assigned to each network are ranked according to the significance of that biological function to the network [57,58].

### 4.9. Death/Apoptosis Assay

MDA-MB-231 cells, non-treated and treated with 0.5 µg/mL of human IgG1 isotype control or atezolizumab, were cultured in 48-well tissue culture-treated plates (0.25 × 10^6^ cells per well) for five days. Cells were collected and washed in cold staining buffer, and stained with Annexin V- Fluorescein isothiocyanate (FITC)and 7-AAD according to the manufacturer’s protocol (Biolegend). Apoptotic and necrotic cells were determined by flow cytometry. Data were acquired by BD LSRFortessa X-20 flow cytometer (BD Biosciences), and analyzed by FlowJo v.10.0 software (Tree Star).

### 4.10. BrdU Cell Proliferation Assay

To examine the effect of atezolizumab on MDA-MB-231 cell proliferation, a BrdU cell proliferation chemiluminescent assay kit was utilized (Cell Signaling Technology). This assay detects BrdU incorporation into cellular DNA during cell proliferation. On day 0, MDA-MB-231 cells, non-treated and treated with 0.5 µg/mL of human IgG1 isotype control or atezolizumab, were cultured for five days in a black 96-well tissue culture-treated plate (3000 cells per well). Then BrdU assay was performed according to the manufacture’s protocol. The quantity of BrdU incorporation into cellular DNA was determined by a plate-based luminometer to measure the relative light units (RLU) at 425 nM (Promega Corporation, Madison, WI, USA). Results are expressed as a fold of change in BrdU incorporation.

### 4.11. MTT Cell Viability Assay

The MTT assay was used to examine the cytotoxic effect of atezolizumab on MDA-MB-231 cells. On day 1, MDA-MB-231 cells were plated at a concentration of 0.1 × 10^5^ in a 96-well plate and incubated at 37 °C overnight to allow cell adherence. The following day (day 0), MDA-MB-231 cells were treated with or without 0.5 µg/mL of human IgG1 isotype control or atezolizumab. MTT absorbance was measured on day 0 (before treatment), day 1 (after one dose of Ab), and day 3 (after three doses of Ab). At each time point, 10 µL of MTT (Sigma-Aldrich) per well was added to 100 µL of cell culture medium, followed by a 3 h incubation period. The culture medium was removed and 100 µL of dimethyl sulfoxide (DMSO; Sigma-Aldrich) was added per well. MTT absorbance was measured by a NanoQuant Plate™ reader (TECAN, Männedorf, Switzerland) at 565 nM, using the Microplate reader software i-control™ (TECAN). High absorbance values are positively correlated with cell viability.

### 4.12. Statistical Analysis

All statistical analyses were performed using GraphPad Prism version 8.0 software (GraphPad Software, Inc., San Diego, CA, USA). Normality was checked using the Shapiro–Wilk normality test, and a paired *t*-test was used to determine statistical significance. The *p*-values are represented as the following: ** *p* < 0.01, * *p* < 0.05. A *p*-value of >0.05 was considered statistically non-significant. Data are represented as mean ± standard error of the mean (SEM).

## 5. Conclusions

We elucidated some interesting molecular mechanisms and signaling pathways regulated by atezolizumab in the highly aggressive TNBC cell line. We showed that atezolizumab alters the function and expression profile for MDA-MB-231 cells to limit their proliferative capacity, restrict EMT/metastasis, reduce the induction of hypoxia, and promote DNA repair. Using functional assays, we confirmed that atezolizumab induces cell death/apoptosis and reduces the proliferation of MDA-MB-231 cells.

## Figures and Tables

**Table 1:** Primer sequences for the RT-qPCR.

Primer	Sequence
*PD-L1*	Forward, 5′- TGGCATTTGCTGAACGCATTT -3′
	Reverse, 5′- TGCAGCCAGGTCTAATTGTTTT -3′
*NFKBIB*	Forward, 5′- CGACACCTACCTCGCTCAG -3′
	Reverse, 5′- GTCGGAATCGGGGTACAAGG -3′
*COMMD6*	Forward, 5′- GGAAACTGGGTATGGCTGTGA -3′
	Reverse, 5′- TGTGGAATCGTCATTTCAAAGCA -3′
*BTG2*	Forward, 5′- ACGGGAAGGGAACCGACAT-3′
	Reverse, 5′- CAGTGGTGTTTGTAGTGCTCTG -3′
*RNF122*	Forward, 5′- ATTCCAGTGGTGTAACGGGTG -3′
	Reverse, 5′- CCTGTGCCGAAGATGACCATA -3′
*NBL1*	Forward, 5′- CATGTGGGAGATTGTGACGCT-3′
	Reverse, 5′- CCTCGTGACTAGGCTCCTTG -3′
*ICAM3*	Forward, 5′- GGAGTTCCTTTTGCGGGTG -3′
	Reverse, 5′- TCAGAGCTGGGACAATCAGTA -3′
*TRAF5*	Forward, 5′- CCACTCGGTGCTTCACAAC -3′
	Reverse, 5′- GTACCGGCCCAGAATAACCT -3′
*SNAI1*	Forward, 5′-TCGGAAGCCTAACTACAGCGA -3′
	Reverse, 5′- AGATGAGCATTGGCAGCGAG -3′
*VIM*	Forward, 5′- GACGCCATCAACACCGAGTT-3′
	Reverse, 5′- CTTTGTCGTTGGTTAGCTGGT-3′
*CDH1*	Forward, 5′- CGAGAGCTACACGTTCACGG -3′
	Reverse, 5′- GGGTGTCGAGGGAAAAATAGG -3′
*β-ACTIN*	Forward, 5′- AGAGCTACGAGCTGCCTGAC -3′
	Reverse, 5′- AGCACTGTGTTGGCGTACAG -3′

## Supplementary Materials

The following are available online at https://www.mdpi.com/2072-6694/11/8/1050/s1, Figure S1: Detailed information of protein expression analysis by Western blot, Figure S2: KEGG pathway analysis of upregulated genes in MDA-MB-231 treated cells showing the effect of atezolizumab on genes involved in P53 pathway.
