# Supplementary material for: PD-L1 Blockade by Atezolizumab Downregulates Signaling Pathways Associated with Tumor Growth, Metastasis, and Hypoxia in Human Triple Negative Breast Cancer
Source: Cancers (Basel). 2019 Jul 25;11(8):1050. doi: 10.3390/cancers11081050 (PMC6721435; doi:10.3390/cancers11081050)
Supplement: Supplementary file 1 [file cancers-11-01050-s001.pdf]

*Supplementary Materials*

# PD-L1 Blockade by Atezolizumab Downregulates Signaling Pathways Associated with Tumor Growth, Metastasis, and Hypoxia in Human Triple Negative Breast Cancer

**Reem Saleh, Rowaida Z. Taha, Varun Sasidharan Nair, Nehad M. Alajez and Eyad Elkord**

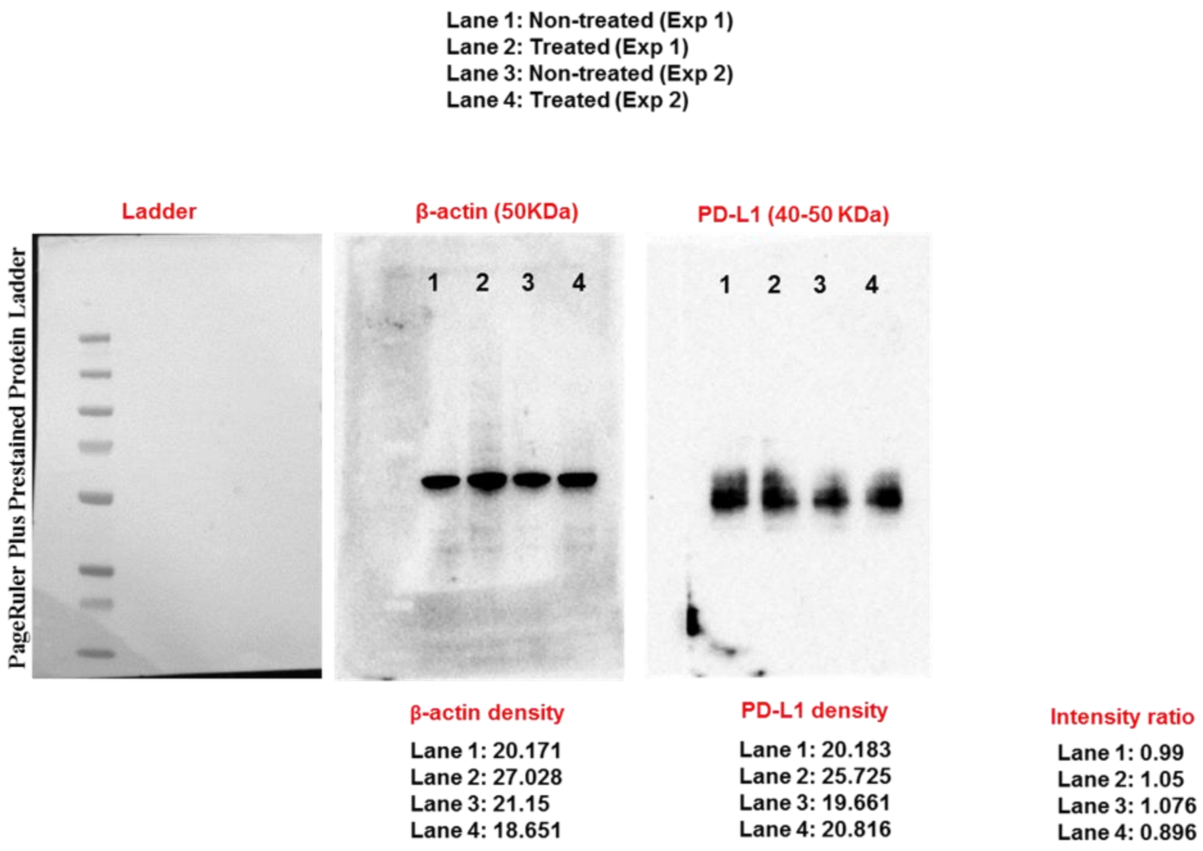


**Figure S1.** Detailed information of protein expression analysis by Western blot.

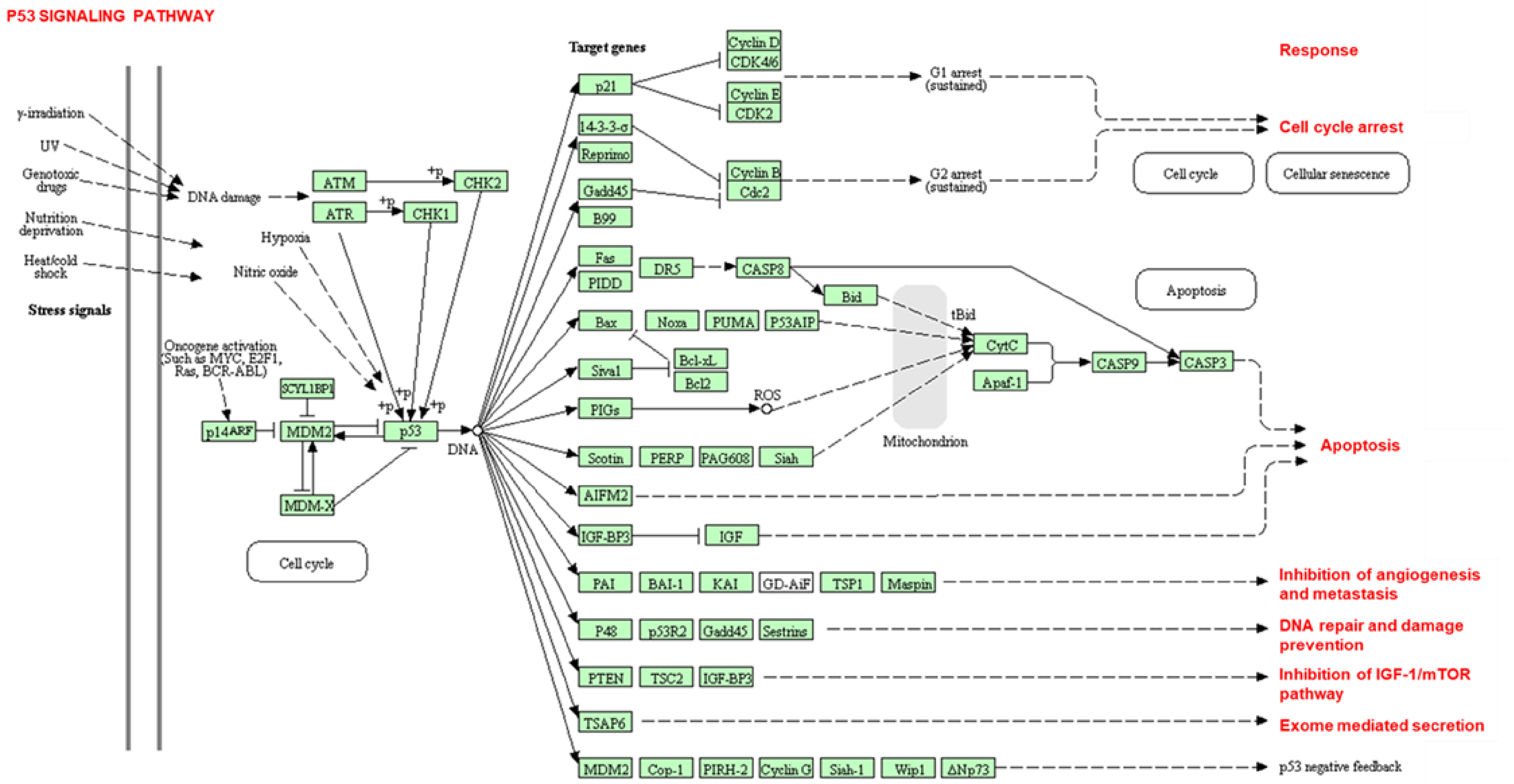


**Figure S2.** KEGG pathway analysis of upregulated genes in MDA-MB-231 treated cells showing the effect of atezolizumab on genes involved in P53 pathway.

© 2019 by the authors. Licensee MDPI, Basel, Switzerland. This article is an open access article distributed under the terms and conditions of the Creative Commons Attribution (CC BY) license (http://creativecommons.org/licenses/by/4.0/).
